# Can cholesterol be used to distinguish pleural exudates from transudates? evidence from a bivariate meta-analysis

**DOI:** 10.1186/1471-2466-14-61

**Published:** 2014-04-15

**Authors:** Yongchun Shen, Hong Zhu, Chun Wan, Lei Chen, Tao Wang, Ting Yang, Fuqiang Wen

**Affiliations:** 1 Division of Pulmonary Diseases, State Key Laboratory of Biotherapy of China and Department of Respiratory and Critical Care Medicine, West China Hospital of Sichuan University, Chengdu 610041, China; 2 Department of Abdominal Cancer, West China Hospital of Sichuan University, Chengdu 610041, China

**Keywords:** Cholesterol, Exudate, Pleural fluid, Meta-analysis

## Abstract

**Background:**

Many studies have investigated whether pleural cholesterol levels can aid in diagnosis of pleural exudates, and the results have varied considerably. To gain a more reliable answer to this question, we meta-analyzed the literature on using pleural cholesterol or the ratio of cholesterol in pleural fluid to cholesterol in serum (P/S cholesterol ratio) as diagnostic tests to help identify pleural exudates.

**Methods:**

Literature databases were systematically searched for studies examining accuracy of pleural cholesterol or P/S cholesterol ratios for diagnosing pleural exudates. Data on sensitivity, specificity, positive/negative likelihood ratio (PLR/NLR), and diagnostic odds ratio (DOR) were pooled using bivariate-effects models. Summary receiver operating characteristic (SROC) curves and area under the curve (AUC) were used to summarize overall test performance.

**Results:**

Our meta-analysis included up to 20 studies involving 3,496 subjects. Summary estimates for pleural cholesterol in the diagnosis of pleural exudates were as follows: sensitivity, 0.88 (95%CI 0.84 to 0.92); specificity, 0.96 (95% CI 0.92 to 0.98); PLR, 20.31 (95% CI 11.21 to 36.78); NLR, 0.12 (95% CI 0.09 to 0.17); DOR, 167.06 (95% CI 76.79 to 363.95); and AUC 0.97 (95% CI 0.95 to 0.98). The corresponding summary performance estimates for using the P/S cholesterol ratio were as follows: sensitivity, 0.94 (95% CI 0.92 to 0.96); specificity, 0.87 (95% CI 0.83 to 0.91); PLR 7.46 (95% CI, 5.47 to 10.19); NLR, 0.07 (95% CI 0.05 to 0.10); DOR, 107.74 (95% CI 60.91 to 190.60); and AUC 0.97 (95% CI 0.95 to 0.98).

**Conclusions:**

Both pleural cholesterol level and the P/S cholesterol ratio are helpful for the diagnosis of pleural exudates. Nevertheless, the results of pleural cholesterol assays should be interpreted in parallel with the results of traditional tests and clinical information.

## Background

Pleural effusion often develops in patients with thoracic or systemic diseases [1]. Such effusion has traditionally been classified as transudate or exudate based on the etiology and underlying pathology, and differentiating the two types of pleural effusion is critical for guiding treatment [2,3]. Transudates are usually taken as a sign of underlying congestive heart failure, cirrhosis, or nephrosis, which then becomes the focus of treatment. Because in the case of a transudate, aetiology and therapy are directed to the underlying congestive heart failure, cirrhosis, or nephrosis. Alternatively, exudates are usually taken as a sign of, inflammatory disorders or malignancy, leading to more extensive diagnostic procedures [4,5].

The criteria most widely used to differentiate exudates and transudates in patients with pleural effusions are Light's criteria, established by Light et al. in 1972 [6]. These criteria rely on levels of total protein and of lactate dehydrogenase [6]. The criteria maximize sensitivity at the expense of specificity: they typically identify 98% of pleural exudates, but they misclassify approximately 25% of transudates as exudates [7]. As a result, patients misclassified with exudate undergo unnecessary and risky invasive diagnostic procedures, such as thoracoscopic pleural biopsy and image-guided percutaneous pleural biopsy [8]. This highlights the need to develop better methods to differentiate exudative and transudative pleural effusions [9].

Since Light's criteria were published in 1972, pleural cholesterol levels have been explored for their usefulness in diagnosing pleural exudates. Cellular degeneration and vascular leakage due to increased permeability are thought to elevate pleural cholesterol levels [10]. Several studies suggest that pleural cholesterol is increased in pleural exudates, making it a potential biomarker for differentiating exudative and transudative pleural effusions [11]. Studies have come to conflicting conclusions about whether pleural cholesterol levels can provide adequate differentiating power, and other studies have explored the diagnostic potential of the ratio of cholesterol in pleural fluid to cholesterol in serum (P/S cholesterol ratio) and come to similarly conflicting conclusions [11]. To help gain more reliable insights, we meta-analyzed the literature on using pleural cholesterol level or the P/S cholesterol ratio to distinguish pleural exudates from transudates.

## Methods

The present meta-analysis was performed using the guidelines of the Preferred Reporting Items for Systematic Reviews, as well as the Meta-analysis Statement and methods recommended by the Cochrane Diagnostic Test Accuracy Working Group [12]. Institutional review board approval was not required for this retrospective meta-analysis.

Two investigators (YCS and HZ) searched in PUBMED and EMBASE for relevant articles published up to October 2013 in which the following search terms were used as Medical Headings and/or text words: “cholesterol”, “pleural effusion”, and “pleural fluid”. The syntax for the PUBMED searches was as follows: “pleural effusion” OR “pleural fluid” AND “cholesterol”. Reference lists of the included studies and review articles were also checked to identify additional studies.

Studies were included if they fulfilled the following criteria: (1) they were original research articles published in English; (2) they examined the ability of pleural cholesterol level or P/S cholesterol ratio for differentiating pleural transudates and exudates in humans; and (3) they reported sufficient data to allow calculation of true positive (TP), false positive (FP), false negative (FN), and true negative (TN) rates. Conference proceedings and studies published only as abstracts were excluded. To avoid selection bias, we also excluded studies involving fewer than 20 patients.

Two reviewers (LC and TW) independently identified eligible studies and extracted data, with which they prepared 2 × 2 tables of diagnostic performance. In case of disagreement, the two reviewers arrived at a consensus. The quality of the selected studies was assessed using the 14-items Quality Assessment of Diagnostic Accuracy Studies (QUADAS) list [13].

Using bivariate regression, we calculated pooled estimates of sensitivity (SEN) and specificity (SPE) as the main outcome measures, and we constructed summary receiver operating characteristic (SROC) curves [14]. The bivariate approach investigates potential between-study heterogeneity and takes into account possible correlation between SEN and SPE. Based on the pooled estimates of SEN and SPE, we calculated positive likelihood ratios (PLR) and negative likelihood ratios (NLR). SEN and SPE estimates were paired to generate diagnostic odds ratios (DOR), which we used as an overall index of diagnostic accuracy. DOR relates the odds of positive test results in those with the condition with the odds of positive test results in those without the condition.

Heterogeneity was assessed using the I^2^ inconsistency test. I^2^ > 50% indicated substantial heterogeneity, which was then analyzed through subgroup analyses. The following covariates were clearly reported by more than 80% of included studies and so were analyzed as possible sources of heterogeneity: publication year (before 2000 vs. after 2000), design (cross sectional vs. non-cross sectional), data collection (prospective vs. retrospective), sampling method (consecutive/random vs. nonconsecutive/nonrandom/not reported), blinding (yes vs. no or not reported), detection method (enzymatic colorimetric method vs. other), sample size (<100 subjects vs. ≥ 100 subjects), QUADAS score (<9 vs. ≥9), and cholesterol cut-off value (60 mg/dl vs. other) [11].

Deeks’s funnel plot was used to detect publication bias [15]. Post-test probability (PTP) was calculated using the overall prevalence of 20% with Fagan nomograms. All analyses were performed using the “Midas” module in STATA 12.0 (Stata Corp., College Station, TX). All statistical tests were two-sided, with P values less than 0.05 taken as the threshold for statistical significance.

## Results

**Figure 1:**
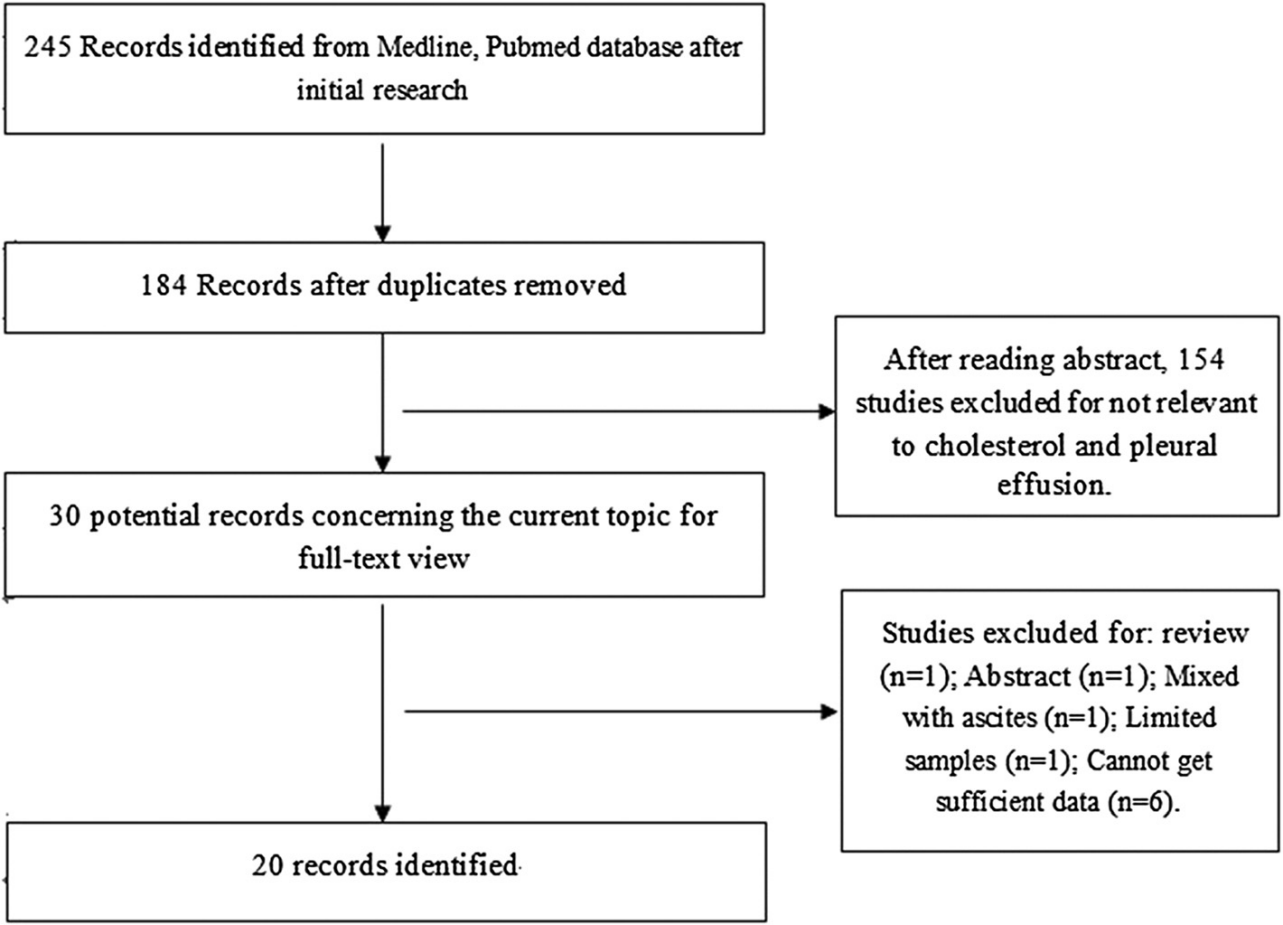
Flow diagram of study selection.

After systematically searching literature databases and manually searching references lists in relevant reviews and studies, we included 20 studies examining the diagnostic accuracy of cholesterol levels in patients with pleural exudates [16-35]. Studies were excluded mainly because they did not examine diagnostic performance, they did not report sufficient data to construct 2 × 2 tables, or they involved fewer than 20 patients. Figure 1 outlines the process of selecting studies.

### Quality of reporting and study design

The final set of 20 studies involved 3,496 subjects, comprising 2,548 patients with pleural exudates and 948 with transudates. Of the included studies, 19 examined the ability of pleural cholesterol concentrations to distinguish exudates from transudates, while 13 examined the ability of the P/S cholesterol ratio to do this. Included studies were published between 1987 and 2013. In all included studies, most studies took into account the limitations of Light's criteria and therefore diagnosed exudative pleural effusion by combining Light's criteria with clinical information, treatment response, and other data, which is widely considered an acceptable basis for exudate diagnosis. Of the 20 studies, 12 had QUADAS scores ≥9. Tables 1 and 2 summarize the clinical characteristics of the patients in each study as well as the QUADAS scores for each publication.

**Table 1:** Clinical summary of included studies examining the diagnostic accuracy of pleural cholesterol level

**Study (ref no.)**	**Year**	**Country**	**No. patients**		**Standard**	**Method**	**Cut-off (mg/l)**	**TP**	**FP**	**FN**	**TN**	**QUADAS score**	Misclassified*
			**Exu**	**Trans**									
Hamm H [16]	1987	Germany	31	31	Clinical criteria	ECM	60	28	3	3	28	8	9/30
Valdés L [17]	1991	Spain	188	65	Clinical criteria	ECM	55	171	0	17	65	9	14/65
Romero S [18]	1993	Spain	253	44	Clinical criteria	ECM	60	206	4	47	40	8	10/44
Burgess LJ [19]	1995	South Africa	270	123	Clinical criteria	ESM	50	184	11	86	112	7	19/112
Costa M [20]	1995	Chile	131	49	Clinical criteria	ECM	45	118	0	13	49	9	9/49
Gil Suay V [21]	1995	Spain	156	48	Clinical criteria	ECM	54	149	4	7	44	8	17/48
Garcia-Pachon E [22]	1996	Spain	118	35	Clinical criteria	NA	50	107	1	11	34	10	9/35
Kalayci AG [23]	1996	Turkey	40	20	Clinical criteria	ECM	40	36	1	4	19	9	NA
Metintaş M [24]	1997	Turkey	72	21	Clinical criteria	ECM	60	54	3	18	18	10	4/21
Gázquez I [25]	1998	Spain	155	38	Clinical criteria	ECM	50	130	6	25	32	7	11/38
Romero S [26]	2000	Spain	182	61	Clinical criteria	ECM	60	155	5	27	56	9	9/61
Yilmaz A [27]	2000	Turkey	150	105	Clinical criteria	ECM	60	143	4	7	101	8	13/105
Porcel JM [28]	2001	Spain	139	32	Clinical criteria	ECM	60	111	1	28	31	9	8/32
Alexandrakis MG [29]	2002	Greece	82	24	Light's criteria	NA	65	76	0	6	24	10	NA
Guleria R [30]	2003	India	50	25	Clinical criteria	NA	60	44	0	6	25	9	5/25
Leers MP [31]	2007	Netherlands	300	108	Clinical criteria	ECM	60	227	2	73	106	11	29/108
Razi E [32]	2008	Iran	70	49	Light's criteria	NA	38	61	10	9	39	8	NA
Hamal AB [33]	2013	Nepal	43	19	Clinical criteria	ECM	45	42	0	1	19	12	NA
Patel AK [34]	2013	India	49	11	Clinical criteria	NA	60	48	0	1	11	11	0/11

Abbreviations: ECM, enzymatic colorimetric method; ESM, enzymatic spectrophotometric method; Exu, pleural exudate; Trans, transudate; TP, no. of true positives; FP, no. of false positives; FN, no. of false negatives; NA, not applicable; TN, no. of true negatives; QUADAS, quality assessment of diagnostic accuracy studies.

*No. of transudates misclassified as exudates based on Light’s criteria (misclassifications/total).

**Table 2:** Clinical summary of included studies examining the diagnostic accuracy of the ratio of cholesterol in pleural fluid to cholesterol in serum

**Study (ref. no.)**	**Year**	**Sample size**		**Cut-off**	**TP**	**FP**	**FN**	**TN**
		**Exudates**	**Transudates**					
Hamm H [16]	1987	31	31	0.3	29	2	2	29
Valdés L [17]	1991	188	65	0.3	174	8	14	57
Romero S [18]	1993	249	42	0.3	222	12	27	30
Burgess LJ [19]	1995	270	123	0.3	240	23	30	100
Gil Suay V [21]	1995	156	48	0.32	152	4	4	44
Garcia-Pachon E [22]	1996	118	35	0.3	109	1	9	34
Kalayci AG [23]	1996	40	20	0.3	38	2	2	18
Metintaş M [24]	1997	72	21	0.3	67	6	5	15
Romero S [26]	2000	182	61	0.3	171	11	11	50
Yilmaz A [27]	2000	150	105	0.3	143	11	7	94
Horvath LL [35]	2001	69	40	0.3	68	2	1	38
Alexandrakis MG [29]	2002	82	24	0.38	77	3	5	21
Guleria R [30]	2003	50	25	0.4	49	4	1	21

Abbreviations: TP, no. of true positives; FP, no. of false positives; FN, no. of false negatives; NA, not applicable; TN, no. of true negatives.

### Diagnostic accuracy of pleural cholesterol level

**Figure 2:**
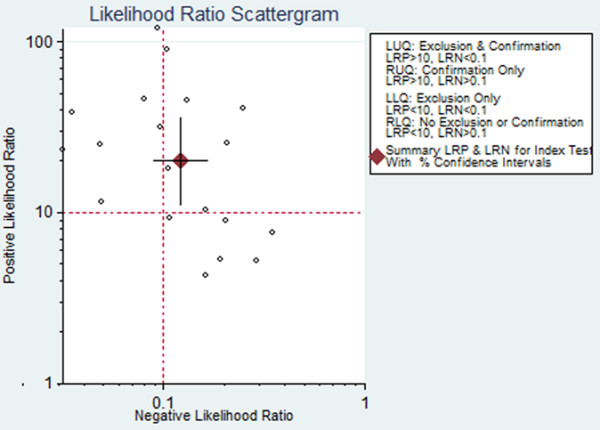
Scatterplot of the positive likelihood ratio (PLR) and negative likelihood ratio (NLR) when using pleural cholesterol concentrations to diagnose exudates.

The following pooled parameters were calculated over all 19 studies examining pleural cholesterol concentrations for diagnosing exudates: SEN, 0.88 (95% CI: 0.84-0.91); SPE, 0.96 (95% CI: 0.92-0.98); PLR, 20.31 (95% CI: 11.21-36.78) (Figure 2); NLR, 0.12 (95% CI: 0.09-0.17) (Figure 2); and DOR, 167.06 (95% CI: 76.69-363.95). All five performance indices showed high I^2^ values: SEN, 91.13%; SPE, 74.31%; PLR, 63.7%; NLR, 91.96%; and DOR, 100% (all P < 0.05). This suggests substantial heterogeneity among the studies.

**Figure 3:**
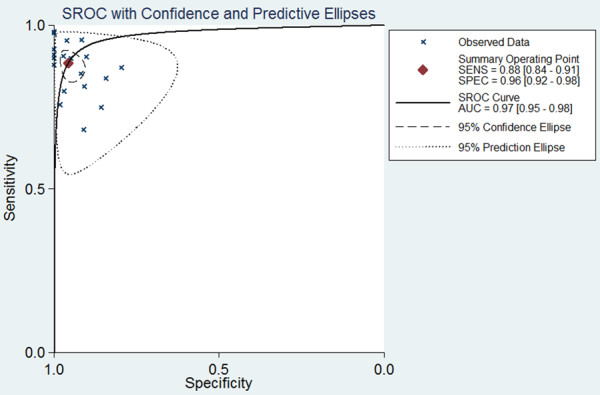
Summary receiver operating characteristic (SROC) curve for pleural cholesterol concentration as a diagnostic test for exudates.

**Figure 4:**
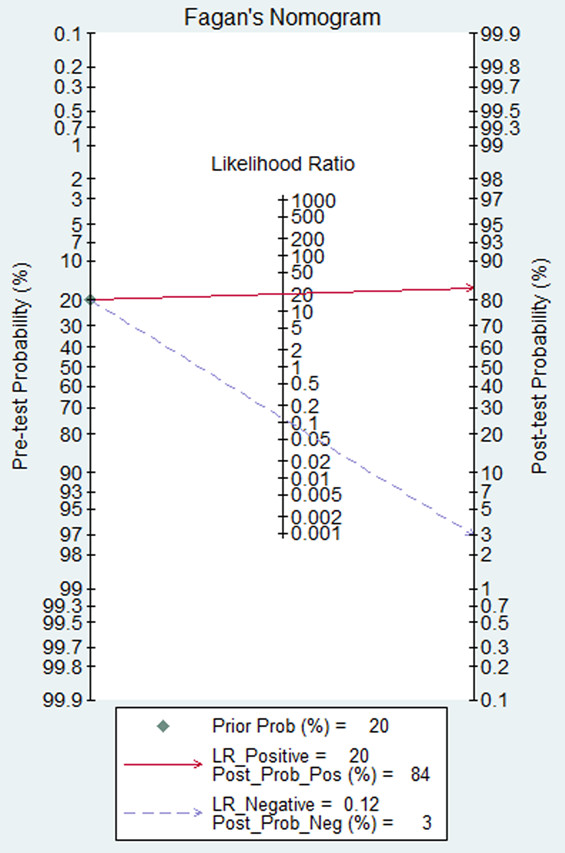
**Fagan**’**s nomogram for likelihood ratios and pre- and post-test probabilities for using pleural cholesterol levels to diagnose pleural exudates.**

Figure 3 shows a plot of the rate of true positives as a function of the rate of false positives for individual studies, as well as the corresponding SROC curve. Using the bivariate approach, which estimates not only the strength but also the shape of the correlation between SEN and SPE, we plotted the observed and predicted ellipses at a 95% confidence level. The AUC was 0.97 (95% CI: 0.95-0.98), indicating a high discriminatory ability of pleural cholesterol. Fagan’s nomogram for likelihood ratios (Figure 4) indicated that using cholesterol to detect pleural exudates increased the post-probability to 83% when the results were positive and reduced the post-probability to 4% when the results were negative.

### Diagnostic accuracy of the P/S cholesterol ratio

**Figure 5:**
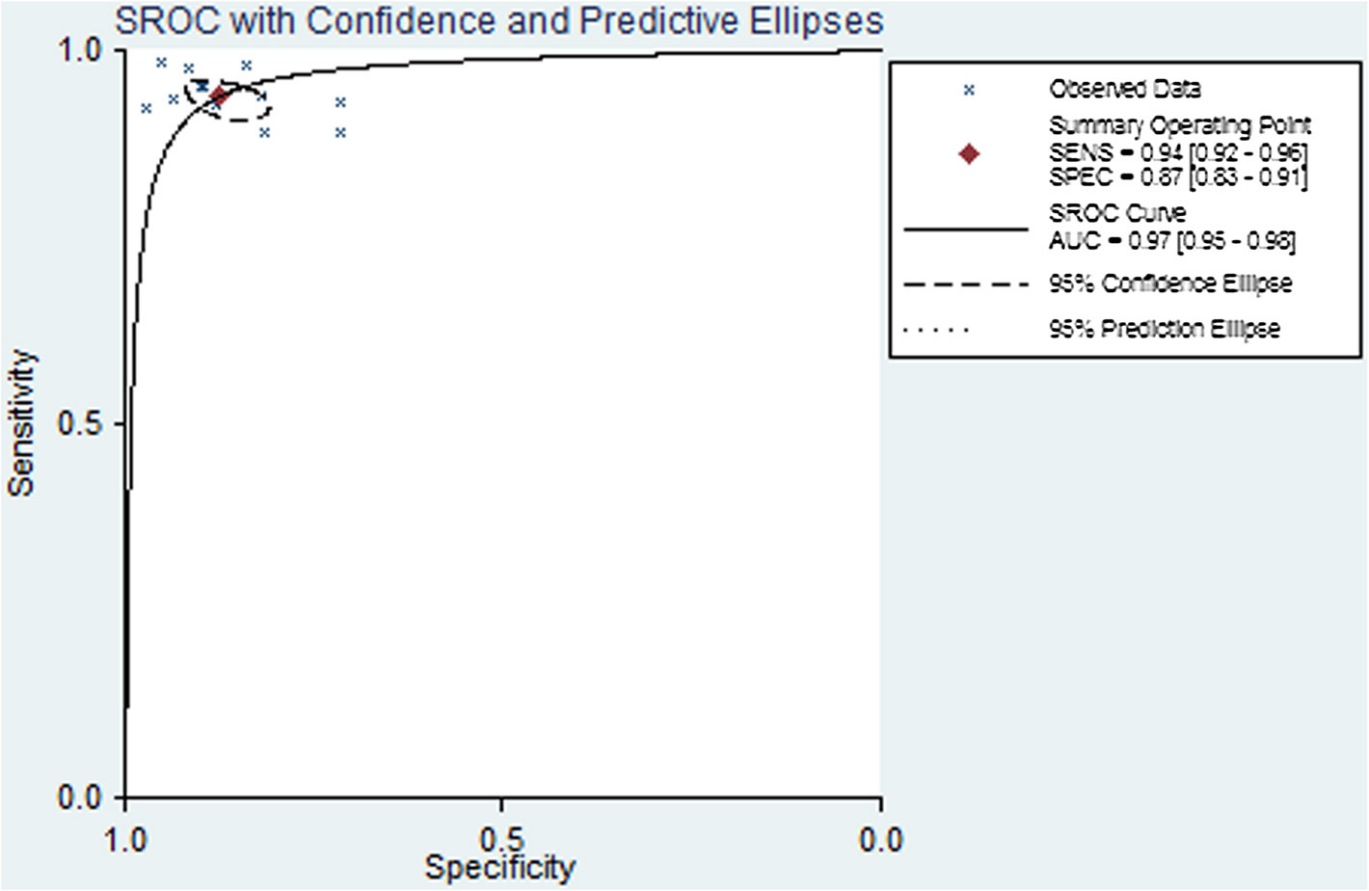
Summary receiver operating characteristic (SROC) curve for using the ratio of cholesterol in pleural fluid to cholesterol in serum to diagnose pleural exudates.

A total of 13 studies with 2,297 subjects examined the ability of the P/S cholesterol ratio to distinguish exudates from transudates. Table 3 summarizes the SEN, SPE, PLR, NLR, and DOR, while Figure 5 shows the SROC curve. The AUC was 0.97 (95% CI: 0.95-0.98), suggesting high overall accuracy.

**Table 3:** Summary characteristics of diagnostic performance of pleural cholesterol levels and ratio of cholesterol in pleural fluid to cholesterol in serum

**Parameter**	**Pleural cholesterol concentrations**	**Pleural fluid to serum cholesterol ratio**
Sensitivity	0.88	0.94
	(95CI: 0.84-0.92)	(95CI: 0.92-0.96)
Specificity	0.96	0.87
	(95CI: 0.92-0.98)	(95CI: 0.83-0.91)
PLR	20.31	7.46
	(95CI: 11.21-36.78)	(95CI: 5.47-10.19)
NLR	0.12	0.07
	(95CI: 0.09-0.17)	(95CI: 0.05-0.10)
DOR	167.06	107.74
	(95CI: 76.69-363.95)	(95CI: 60.91-190.60)
PPP	84%	65%
PPN	3%	2%
AUC	0.97	0.97
	(95CI: 0.95-0.98)	(95CI: 0.95-0.98)

### Investigations of heterogeneity and publication bias

**Figure 6:**
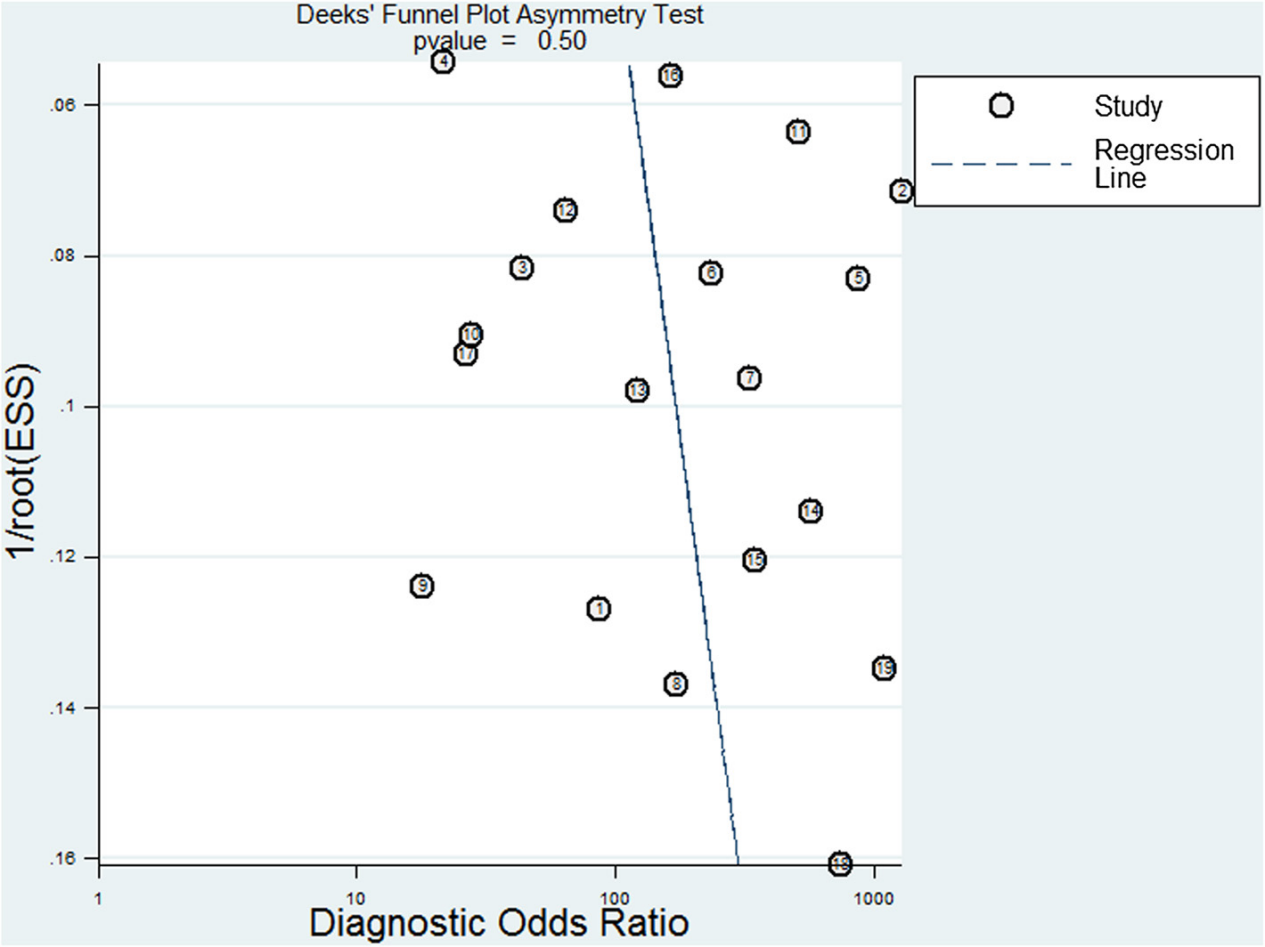
Deek’s funnel plot to assess the likelihood of publication bias.

Significant heterogeneity was identified among included studies, so we performed subgroup analysis to investigate the possible sources of this heterogeneity. Table 4 summarizes the influence of certain covariates on the SEN and SPE. SPE was significantly higher with some covariates, such as QUADAS score or blinding. However, these covariates did not significantly affect SEN. Deeks’ funnel plot asymmetry test was used to assess likelihood of publication bias in the final set of studies. The slope coefficient was associated with a P value of 0.50, suggesting symmetry in the data and low likelihood of such bias (Figure 6).

**Table 4:** Subgroup analysis for sensitivity and specificity

**Covariate**	**Sensitivity**	**Coefficient**	**Z**	**P**	**Specificity**	**Coefficient**	**Z**	**P**
Publication year	0.90 (95% CI 0.84-0.94)	2.19	0.84	0.40	0.97 (95% CI 0.92-0.99)	3.39	0.93	0.35
Method	0.88 (95% CI 0.83-0.92)	2.01	−0.20	0.84	0.96 (95% CI 0.92-0.98)	3.07	−0.10	0.92
Cut-off value	0.86 (95% CI 0.80-0.91)	1.85	−0.96	0.34	0.96 (95% CI 0.91-0.98)	3.08	−0.02	0.99
QUADAS score	0.89 (95% CI 0.84-0.93)	2.07	0.34	0.74	0.98 (95% CI 0.95-0.99)	3.77	3.15	0.00
Sample size	0.87 (95% CI 0.82-0.91)	1.92	−1.03	0.30	0.95 (95% CI 0.91-0.98)	3.02	−0.36	0.72
Prospective	0.90 (95% CI 0.85-0.93)	2.18	1.08	0.28	0.95 (95% CI 0.90-0.98)	2.95	−0.64	0.52
Design	0.90 (95% CI 0.86-0.93)	2.22	1.70	0.09	0.94 (95% CI 0.90-0.97)	2.81	−1.42	0.16
Consecutive	0.87 (95% CI 0.82-0.91)	1.90	−1.50	0.13	0.95 (95% CI 0.91-0.97)	2.87	−1.56	0.12
Blinding	0.88 (95% CI 0.80-0.93)	1.99	−0.16	0.88	0.98 (95% CI 0.95-0.99)	3.95	2.00	0.05

## Discussion

Despite the importance of defining the type of pleural effusion accurately, current methods lack the power to reliably differentiate pleural exudates from transudates [7]. Several studies suggest that pleural cholesterol levels and the P/S cholesterol ratio may be able to accomplish this, but the studies have given conflicting results. Our meta-analysis suggests that both pleural cholesterol level and P/S cholesterol ratio are useful as tools in the diagnosis of pleural exudates, though they probably cannot stand on their own and so should be used in conjunction with more traditional tests.

Our meta-analysis showed that pleural cholesterol level was associated with high SEN (0.88, 95% CI: 0.84-0.91) and SPE (0.96, 95% CI: 0.92-0.98). These findings suggest that pleural cholesterol may represent a new milestone in pleural exudate diagnosis. The SROC curve illustrates overall test performance, and shows the trade-off between SEN and SPE. Our SROC analysis showed an AUC of 0.97, suggesting high overall accuracy. Another indicator of diagnostic accuracy is DOR, which is the ratio of the odds of a true positive to the odds of a false positive; DOR combines SEN and SPE data into a single number ranging from 0 to infinity, with higher values indicating better discriminatory test performance. Mean DOR in our meta-analysis was 167.06, suggesting that assaying pleural cholesterol levels should be helpful in the diagnosis of pleural exudates.

We further examined the diagnostic accuracy of pleural cholesterol levels by calculating PLR and NLR, which can be easier to relate to clinical practice than SROC and DOR [36]. The pooled PLR value of 20.31 suggests that patients with pleural exudates have an approximately 20-fold higher chance of giving a positive pleural cholesterol test result than do patients without exudates. At the same time, the pooled NLR was 0.12, indicating that a negative pleural cholesterol test result is 12% likely to be a false negative, which is not low enough to rule out pleural exudates. This comprehensive analysis of the diagnostic accuracy of pleural cholesterol levels suggests that this indicator may not be reliable enough on its own but should instead be used in conjunction with other conventional tests. This unreliability may in part reflect the sensitivity of pleural cholesterol levels to differences in disease conditions, patient population, and environment.

Using the P/S cholesterol ratio instead of absolute cholesterol levels may help eliminate the effects of such factors to aid the diagnosis of pleural exudates. Our results revealed that P/S cholesterol ratio showed even higher SEN than pleural cholesterol levels (0.94, 95% CI: 0.92-0.96); in addition, the AUC was 0.97, suggesting that the P/S cholesterol ratio also shows high discriminatory ability.

Misclassification of transudates as exudates can lead to inappropriate patient management or potentially unnecessary and invasive diagnostic investigations that increase morbidity and health care costs [37]. Therefore we compared how often clinicians misclassified pleural transudates as exudates depending on whether they relied on pleural cholesterol levels or Light’s criteria (Table 1). Pleural cholesterol levels were associated with a significantly lower misclassification rate. We conclude that pleural cholesterol level shows substantial promise as a supplementary test for distinguishing pleural exudates from transudates.

While meta-analysis is well-suited for generating summary outcomes, the pooled results can mask heterogeneity that should be understood in detail [38]. Indeed we detected substantial heterogeneity across the included studies, and subgroup analyses suggest that differences in QUADAS score accounted for most of the observed SPE heterogeneity. Such differences did not, however, significantly affect SEN, suggesting that study quality had little influence on the TP rate of pleural cholesterol tests. Given the effect of study quality on SPE and the low QUADAS scores of several included studies, future studies should aim for greater rigor in order to decrease the risk of bias.

Our meta-analysis suggests an association between elevated pleural cholesterol and the presence of pleural exudates, which implies that cholesterol contributes to exudate pathogenesis. It is not immediately clear how this happens, so future research should examine this question in order to provide a biological basis for the observed association. Our meta-analysis also points out the need for investigating the effect of cut-off value on the diagnostic accuracy of pleural cholesterol levels. The values in our meta-analysis ranged from 38 to 65 mg/dl. This variation in cut-off value partly reflects differences in clinical context: a lower cut-off value is typically used for cardiac patients with pleural effusion than for patients in cancer institutes. Further work should aim to identify the cut-off value that provides optimal diagnostic accuracy [11].

The findings of this meta-analysis should be interpreted with caution because of several limitations. While our strict inclusion and exclusion criteria may have helped reduce selection bias, they led to a relatively small final set of studies for which statistical power may be inadequate for drawing definitive conclusions about the ability of pleural cholesterol levels to discriminate between exudates and transudates. For example, we included only studies published in English in a relatively small number of databases. Our results may be biased by our omission of unpublished studies, studies published in other languages and studies published in journals not indexed in the databases we searched.

## Conclusions

Our meta-analysis suggests that assaying pleural cholesterol levels and the P/S cholesterol ratio may significantly aid the diagnosis of pleural exudates. In the near future, cholesterol-based assays may prove useful as a non-invasive confirmatory test to complement current screening procedures and as a rapid clinical test to guide the comprehensive management of patients with pleural effusion.

## Abbreviations

P/S cholesterol ratio: Ratio of cholesterol level in pleural fluid to cholesterol level in serum; TP: True positive; FP: False positive; FN: False negative; TN: True negative; QUADAS: Quality assessment of diagnostic accuracy studies; SEN: Sensitivity; SPE: Specificity; SROC: Summary receiver operating characteristic; PLR: Positive likelihood ratio; NLR: Negative likelihood ratio; DOR: Diagnostic odds ratio; AUC: Area under the curve.

## Competing interests

All authors declare that they have no conflicts of interest or financial disclosures.

## Authors’ contributions

YCS and HZ: conceived the article and contributed to the systematic review, meta-analysis, and manuscript writing. LC and Tao: contributed to the systematic review and manuscript writing. CW and TY: contributed to the systematic review and manuscript writing. FQW: guarantor of the manuscript who takes responsibility for the integrity of the work as a whole, from inception to published article. All authors read and approved the final manuscript.

## Pre-publication history

The pre-publication history for this paper can be accessed here:

http://www.biomedcentral.com/1471-2466/14/61/prepub

## References

[B1] Fisk M Branley H Pleural effusion Br J Hosp Med 2013 74 C50 C54 10.12968/hmed.2013.74.sup4.c50 23571314

[B2] Light RW Pleural effusions Med Clin North Am 2011 95 1055 1070 10.1016/j.mcna.2011.08.005 22032427

[B3] Sahn SA Getting the most from pleural fluid analysis Respirology 2012 17 270 277 10.1111/j.1440-1843.2011.02100.x 22059482

[B4] Kruger D Evaluating the adult with new-onset pleural effusion JAAPA 2013 26 20 27 23923283 10.1097/01.jaa.0000431507.82701.f7

[B5] McGrath EE Anderson PB Diagnosis of pleural effusion: a systematic approach Am J Crit Care 2011 20 119 127 10.4037/ajcc2011685 21362716

[B6] Light RW Macgregor MI Luchsinger PC Ball WC Jr Pleural effusions: the diagnostic separation of transudates and exudates Ann Intern Med 1972 77 507 513 10.7326/0003-4819-77-4-507 4642731

[B7] Porcel JM Identifying transudates misclassified by Light's criteria Curr Opin Pulm Med 2013 19 362 367 10.1097/MCP.0b013e32836022dc 23508114

[B8] Shen YC Liu MQ Wan C Chen L Wang T Wen FQ Diagnostic accuracy of vascular endothelial growth factor for malignant pleural effusion: A meta-analysis Exp Ther Med 2012 3 1072 1076 22970019 10.3892/etm.2012.514 PMC3438727

[B9] Kummerfeldt CE Chiuzan CC Huggins JT Divietro ML Nestor JE Sahn SA Doelken P Improving the predictive accuracy of identifying exudative effusions Chest 2013 145 586 592 24008773 10.1378/chest.13-1142

[B10] Hillerdal G Chyliform (cholesterol) pleural effusion Chest 1985 88 426 428 10.1378/chest.88.3.426 4028854

[B11] Heffner JE Brown LK Barbieri CA Diagnostic value of tests that discriminate between exudative and transudative pleural effusions Chest 1997 111 970 980 10.1378/chest.111.4.970 9106577

[B12] Leeflang MM Deeks JJ Gatsonis C Bossuyt PM Cochrane Diagnostic Test Accuracy Working Group Systematic reviews of diagnostic test accuracy Ann Intern Med 2008 149 889 897 10.7326/0003-4819-149-12-200812160-00008 19075208 PMC2956514

[B13] Whiting PF Weswood ME Rutjes AW Reitsma JB Bossuyt PN Kleijnen J Evaluation of QUADAS, a tool for the quality assessment of diagnostic accuracy studies BMC Med Res Methodol 2006 6 9 10.1186/1471-2288-6-9 16519814 PMC1421422

[B14] Reitsma JB Glas AS Rutjes AW Scholten RJ Bossuyt PM Zwinderman AH Bivariate analysis of sensitivity and specificity produces informative summary measures in diagnostic reviews J Clin Epidemiol 2005 58 982 990 10.1016/j.jclinepi.2005.02.022 16168343

[B15] Deeks JJ Macaskill P Irwig L The performance of tests of publication bias and other sample size effects in systematic reviews of diagnostic test accuracy was assessed J Clin Epidemiol 2005 58 882 893 10.1016/j.jclinepi.2005.01.016 16085191

[B16] Hamm H Brohan U Bohmer R Missmahl HP Cholesterol in pleural effusions A diagnostic aid. Chest 1987 92 296 302 10.1378/chest.92.2.296 3608600

[B17] Valdés L Pose A Suàrez J Gonzalez-Juanatey JR Sarandeses A San José E Alvarez Dobaña JM Salgueiro M Rodríguez Suárez JR Cholesterol: a useful parameter for distinguishing between pleural exudates and transudates Chest 1991 99 1097 1102 10.1378/chest.99.5.1097 2019164

[B18] Romero S Candela A Martín C Hernández L Trigo C Gil J Evaluation of different criteria for the separation of pleural transudates from exudates Chest 1993 104 399 404 10.1378/chest.104.2.399 8339626

[B19] Burgess LJ Maritz FJ Taljaard JJ Comparative analysis of the biochemical parameters used to distinguish between pleural transudates and exudates Chest 1995 107 1604 1609 10.1378/chest.107.6.1604 7781354

[B20] Costa M Quiroga T Cruz E Measurement of pleural fluid cholesterol and lactate dehydrogenase. A simple and accurate set of indicators for separating exudates from transudates Chest 1995 108 1260 1263 10.1378/chest.108.5.1260 7587426

[B21] Gil Suay V Martínez Moragón E Cases Viedma E Perpiñá Tordera M León Fábregas M Sanchis Aldás J Pleural cholesterol in differentiating transudates and exudates. A prospective study of 232 cases Respiration 1995 62 57 63 10.1159/000196392 7784710

[B22] Garcia-Pachon E Padilla-Navas I Sanchez JF Jimenez B Custardoy J Pleural fluid to serum cholinesterase ratio for the separation of transudates and exudates Chest 1996 110 97 101 10.1378/chest.110.1.97 8681674

[B23] Kalayci AG Gürses N Adam B Albayrak D Significance of pleural fluid cholesterol and beta-2 microglobulin levels for the differentiation of pleural effusions in childhood Clin Pediatr (Phila) 1996 35 353 358 10.1177/000992289603500704 8829005

[B24] Metintaş M Alataş O Alataş F Colak O Ozdemir N Erginel S Comparative analysis of biochemical parameters for differentiation of pleural exudates from transudates Light's criteria, cholesterol, bilirubin, albumin gradient, alkaline phosphatase, creatine kinase, and uric acid Clin Chim Acta 1997 264 149 162 10.1016/S0009-8981(97)00091-0 9293374

[B25] Gázquez I Porcel JM Vives M Vicente De Vera MC Rubio M Rivas MC Comparative analysis of Light's criteria and other biochemical parameters for distinguishing transudates from exudates Respir Med 1998 92 762 765 10.1016/S0954-6111(98)90009-9 9713637

[B26] Romero S Martinez A Hernandez L Fernandez C Espasa A Candela A Martin C Light's criteria revisited: consistency and comparison with new proposed alternative criteria for separating pleural transudates from exudates Respiration 2000 67 18 23 10.1159/000029457 10705257

[B27] Yilmaz A Tunaboyu IK Akkaya E Bayramgürler B A comparative analysis of the biochemical parameters used to distinguish between pleural exudates and transudates Respirology 2000 5 363 367 10.1111/j.1440-1843.2000.00276.x 11192547

[B28] Porcel JM Vives M Vicente De Vera MC Cao G Rubio M Rivas MC Useful tests on pleural fluid that distinguish transudates from exudates Ann Clin Biochem 2001 38 671 675 10.1258/0004563011901082 11732649

[B29] Alexandrakis MG Kyriakou D Alexandraki R Pappa KA Antonakis N Bouros D Pleural interleukin-1 beta in differentiating transudates and exudates: comparative analysis with other biochemical parameters Respiration 2002 69 201 206 10.1159/000063620 12097761

[B30] Guleria R Agarwal SR Sinha S Pande JN Misra A Role of pleural fluid cholesterol in differentiating transudative from exudative pleural effusion Natl Med J India 2003 16 64 69 12816183

[B31] Leers MP Kleinveld HA Scharnhorst V Differentiating transudative from exudative pleural effusion: should we measure effusion cholesterol dehydrogenase? Clin Chem Lab Med 2007 45 1332 1338 17663627 10.1515/CCLM.2007.285

[B32] Razi E Moosavi GA Fakharian E Abedi M Relationship of Pleural and Serum Cholesterol and Lipoprotein Levels in Exudative and Transudative Effusions Tanaffos 2008 7 37 43

[B33] Hamal AB Yogi KN Bam N Das SK Karn R Pleural fluid cholesterol in differentiating exudative and transudative pleural effusion Pulm Med 2013 2013 135036 23365740 10.1155/2013/135036 PMC3556870

[B34] Patel AK Choudhury S Combined pleural fluid cholesterol and total protein in differentiation of exudates and transudates Indian J Chest Dis Allied Sci 2013 55 21 23 23798086

[B35] Horvath LL Gallup RA Worley BD Merrill GA Morris MJ Soluble leukocyte selectin in the analysis of pleural effusions Chest 2001 120 362 368 10.1378/chest.120.2.362 11502630

[B36] Akobeng AK Understanding diagnostic tests 2: likelihood ratios, pre- and post-test probabilities and their use in clinical practice Acta Paediatr 2007 96 487 491 10.1111/j.1651-2227.2006.00179.x 17306009

[B37] Bielsa S Porcel JM Castellote J Mas E Esquerda A Light RW Solving the Light's criteria misclassification rate of cardiac and hepatic transudates Respirology 2012 17 721 726 10.1111/j.1440-1843.2012.02155.x 22372660

[B38] Song F Sheldon TA Sutton AJ Abrams KR Jones DR Methods for exploring heterogeneity in meta-analysis Eval Health Prof 2001 24 126 151 10.1177/016327870102400203 11523383

